# Supplementary material for: The Selectivity for Tumor Cells of Nuclear-Directed Cytotoxic RNases Is Mediated by the Nuclear/Cytoplasmic Distribution of p27KIP1
Source: Molecules. 2021 Mar 2;26(5):1319. doi: 10.3390/molecules26051319 (PMC7957890; doi:10.3390/molecules26051319)
Supplement: Supplementary file 1 [file molecules-26-01319-s001.pdf]

Supplementary Material for

The Selectivity for Tumor Cells of Nuclear-Directed Cytotoxic RNases Is Mediated by the Nuclear/Cytoplasmic Distribution of p27^{KIP1}

Glòria García-Galindo [1], Jessica Castro [1,2], Jesús Matés [1,2], Marlon Bravo [1,2], Marc Ribó [1,2], Maria Vilanova [1,2,*] and Antoni Benito [1,2,*]

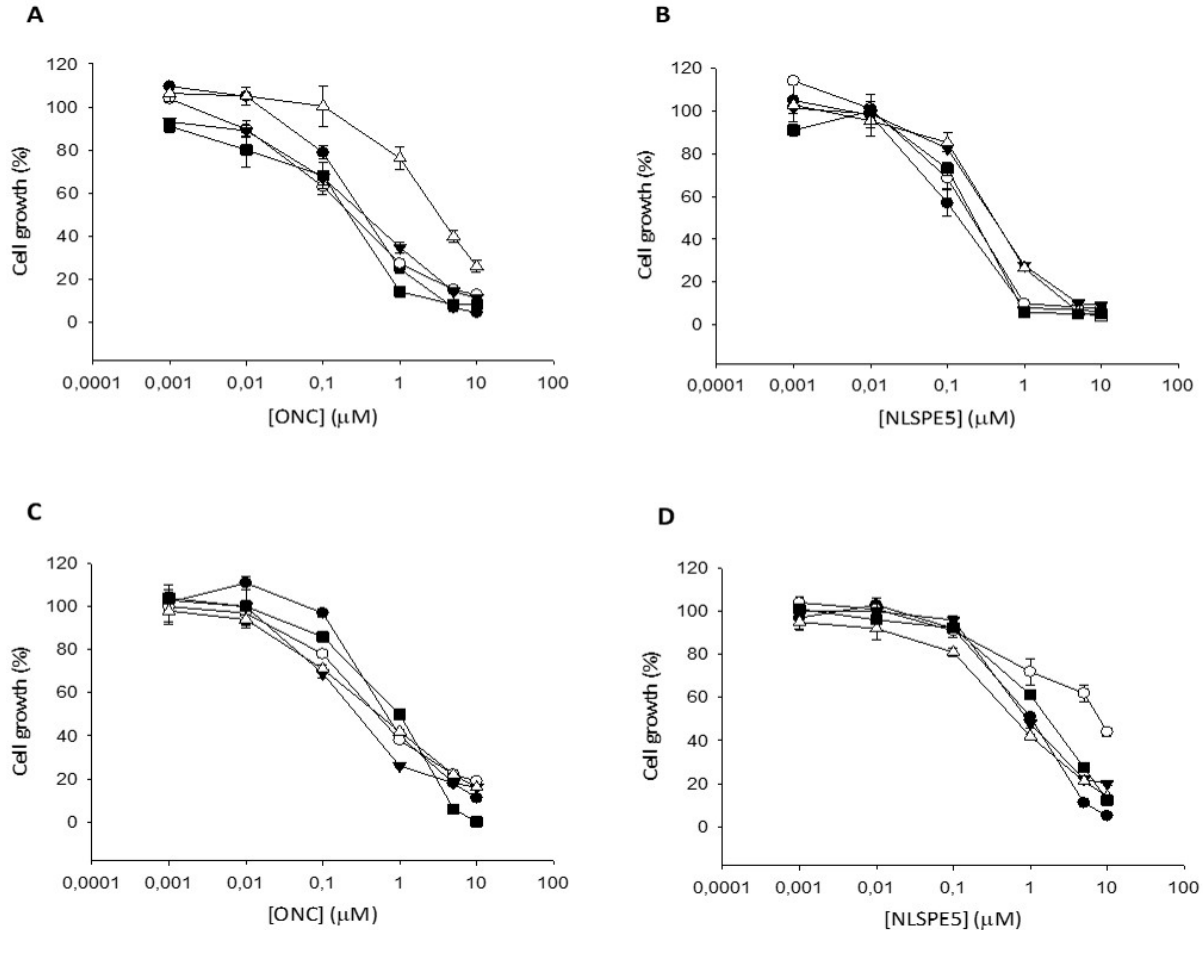

Figure S1. Determination of the cell proliferation of tumor (A and B) and non-tumor (C and D) cell lines treated with onconase (A and C) and NLSPE5 (B and D) determined by the MTT assay. The tumor cell lines are OVCAR-8 (○), NCI-H460 (●), SK-Br-3 (Δ), NCI-H460/R (▼), and HeLa (■); the normal cell lines are CCD-18Co (○), HaCaT (●), HEK-293 (▼), MCF10A (■), and 1BR3G (Δ). Cell growth was measured as indicated in the Materials and Methods section and expressed as the percentage of growth of control cells. The curves in the figure are from one representative experiment. Equivalent results were found in at least three independent experiments.

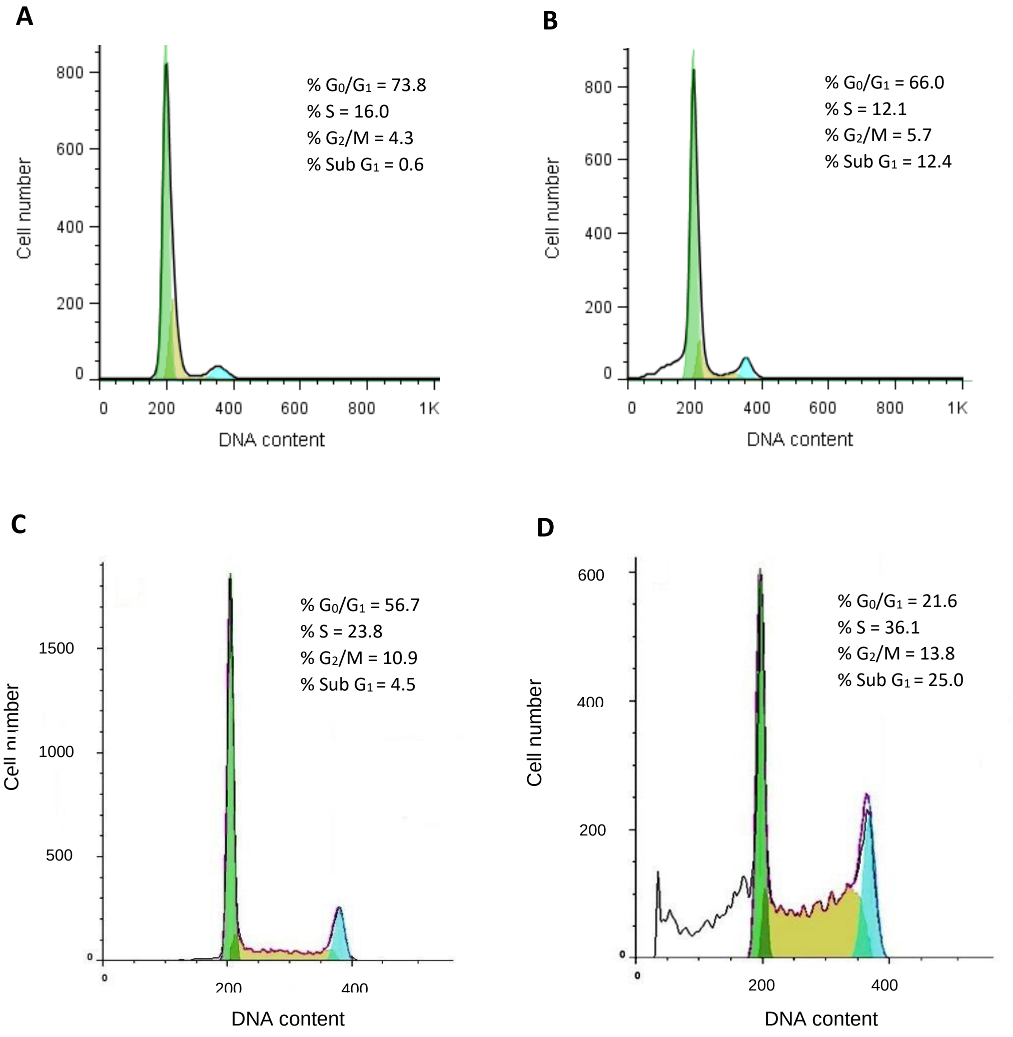

Figure S2. Representative figures of the effects on the cell cycle phase distribution of CCD-18Co (A and B) and NCI-H460/R (C and D) after 72 h of NLSPE5 treatment measured by flow cytometry. Untreated cells (A and C) and NLSPE5 treated cells (B and D) were analyzed as indicated in the Materials and Methods section. Values were analyzed from 10,000 total events.
